# Effects of Substituting Wheat with Waxy Barley Bran Flour on Physical Properties, Health Functionality, and Sensory Characteristics of Noodles

**DOI:** 10.3390/foods14030436

**Published:** 2025-01-28

**Authors:** Yoko Tsurunaga, Ayane Uno, Tetsuya Takahashi, Tsugumi Furuichi

**Affiliations:** 1 Faculty of Human Science, Shimane University, Matsue 690-8504, Japantakahashi@hmn.shimane-u.ac.jp (T.T.); 2 Department of Living Science, Food Science and Nutrition, Tottori College, Kurayoshi 682-8555, Japan; furuichi@cygnus.ac.jp

**Keywords:** waxy barley bran, β-glucan, physical properties, antioxidant activities, sensory evaluation, noodles

## Abstract

When waxy barley (WB) is milled, 40% of the weight is typically discarded as bran. To utilize WB bran resources and improve health functionality, flours prepared from inner bran (IB) and outer bran (OB) layers were used to substitute partially wheat all-purpose flour (APF) for noodle preparation. The dough and noodle qualities were investigated based on analytical tests and sensory evaluations. Both methods revealed considerable darkening of the doughs and noodles upon OB substitution. Boiled noodles with 30% and 50% IB substitution had considerably lower total energy and breaking stress, whereas those with OB substitution had higher breaking stress at all substitution rates. Texture differences between sample groups were observed using analytical tests, but not via sensory evaluation. In addition, the boiled noodles with 50% OB demonstrated considerably lower taste preference in the sensory evaluation than the APF noodles. The comprehensive evaluation score was considerably lower for the boiled noodles with 30% or 50% OB than that of the APF noodles. The β-glucan and antioxidant contents increased with the IB or OB substitution rates. These findings show that APF can be substituted with IB at a substitution rate of 50%, while the substitution of OBF is limited to ≤10%.

## 1. Introduction

Waxy barley (WB) has attracted considerable attention recently because of its high-health functionality and palatability. The main health functionality of WB is its cholesterol-lowering effect, which is related to its high content of β-glucan, a type of soluble dietary fiber [1,2,3,4]. Many countries around the world have verified health claims related to the effects of β-glucan on blood cholesterol, an important biomarker for cardiovascular disease. These findings have led to an increased scientific interest in the research and development of β-glucan-containing foods [5].

WB is often consumed as a pounded grain after the hull and bran are removed. The chewy and unique texture of pounded grains is extensively accepted by consumers; accordingly, they have thus become a popular food product. In particular, the domestic self-sufficiency rate of WB is growing rapidly in Japan, increasing from 3% in 2016 to 49% in 2021, and this growth is expected to continue [6]. Increased production of WB as a pounded grain is expected to increase the amount of outer hull (bran) that is discarded. The pounding yield of WB is approximately 60%, and the remaining 40% in the form of bran is typically used as a fertilizer or discarded [4]. This constitutes a major waste.

There are many reports on the functional health properties of grain bran. For example, rats that were fed whole grains or bran showed a decrease in serum insulin 30 min after eating [7], and barley bran reduced total serum cholesterol in hypercholesterolemic patients [8]. The polyphenolic components in barley bran can inhibit the oxidation of linoleic acid and trap superoxide [9]. Furuichi et al. found that WB bran has considerably higher levels of minerals (K, Mg, Fe, Zn, Mn), a higher total polyphenol content (TPC), and exhibits higher antioxidant activity than polished WB, when evaluated by hydrophilic oxygen radical absorbance capacity (H-ORAC) and 2,2-diphenyl-1-picrylhydrazyl (DPPH) methods [4]. They also found that WB bran contains 3.9–4.8 g/100 g of β-glucan [4].

Thus, wheat noodles with added WB bran, an easily available by-product, are considered to be rich in compounds that enhance functional health. Studies have been conducted on the addition of wheat bran—which has improved functional properties—to noodles. Chen et al. found that adding wheat bran to dry white Chinese noodles increased the fiber content of the noodles [10]. Ling et al. reported that adding 0.25–1.0% arabinoxylans (a key component of wheat bran) to wheat flour enhances both the nutritional value and quality of Chinese noodles [11]. Some reports have indicated that the addition of polysaccharides is effective in improving noodle quality. The addition of carboxymethylcellulose gas to millet pomace-based functional pasta reportedly improved cooking quality characteristics, such as water absorption [12]. The addition of xanthan gum and guar gum to gluten-free pasta improved quality and texture characteristics [13]. In rice noodles, such as rice vermicelli and pho, polysaccharides such as starch can be used to form the noodle band [14]; thus, it is plausible that polysaccharides improve noodle quality. Considering this, β-glucan in WB bran can hypothetically promote noodle band formation. However, WB bran addition may inhibit gluten formability, resulting in poor noodle texture.

It is estimated that approximately 925 million people in the world are malnourished. Meanwhile, in Japan, it is estimated that 24.02 million tons of food waste is generated each year in the agricultural production and food manufacturing industries. Accordingly, there is an urgent need to develop technologies for the effective use of food waste. The manufacturing of high-quality, high-function noodles with functionality equivalent to those of barley bran, a by-product of food waste, would contribute to the realization of a sustainable growth society that supports recycling-oriented practices and global food supply. However, to this day, barley has been mainly used to make beer, shochu, and barley tea, and the amount of bran that is discarded is small; therefore, the parts with the husk have not been sufficiently researched.

In this study, we prepared wheat noodles with added WB bran and evaluated antioxidant activities, total polyphenol, and β-glucan serving as health functionality indicators. Specifically, WB bran used the outer bran (OB) and inner bran (IB) by-products that are produced when polishing WB and produced wheat noodles in which 10, 30, or 50% of the wheat flour was replaced by OB or IB. Moreover, we measured dough extensibility, gluten content (wet gluten amount), color, and texture, and conducted sensory evaluation for quality assessments.

## 2. Materials and Methods

### 2.1. Raw Materials

In this study, the “kirarimochi” variety of the two-row naked WB, which was registered on 28 December 2012 by the National Agriculture and Food Research Organization, was used [6]. Kirarimochi WB was obtained from a grower (Magokoronojo Hirose Agricultural Union Corporation) in the Tottori City in Japan. The WB grains were milled using a barley milling machine (3RSB-10; Hohden Industry Co., Ltd., Kameoka City, Japan). The time required to mill 10 kg of raw barley was approximately 4 h. The hull that emerged during the first 2 h was considered the outer bran flour (OBF), and that obtained during the last 2 h was the same as the inner bran flour (IBF). Other ingredients included commercially available all-purpose flour (APF) (Nisshin Seifun Welna Inc., Chiyoda-ku, Japan) and salts.

### 2.2. Particle-Size Measurements

The particle-size distributions of the samples were analyzed using the method described by Taniguchi et al. [15] with slight modifications. A laser scattering particle size analyzer (Partica LA-960V2, Horiba, Kyoto City, Japan) was used for the measurements. Ethanol (99.5%) (Nacalai Tesque Corporation, Nakagyo-ku, Japan) was used as the dispersant. The samples were added directly to ethanol without suspension, and ultrasonic treatment was not performed. The distribution curve, volume average particle diameter, and D_50_ were measured to determine the particle-size distribution of the samples.

### 2.3. Noodle Preparation

In addition to the flour, salt (9 g) and water (141 g) were used to prepare the noodles. In the control groups, 300 g of APF was used. In the test groups, a part of APF was substituted by IBF or OBF. Specifically, 10% (30 g), 30% (90 g), or 50% (150 g) of APF was substituted by IBF or OBF to produce the dough and the noodles. The substitution rate of both IBF and OBF was limited to 50% because preliminary tests confirmed that kneading is extremely difficult when rates >50% are used. The test groups in which part of the APF was replaced with IBF or OBF also used salt (9 g) and water (141 g) to make noodles. A kneader (PK601, Nippon Kneader Co., Kanagawa, Japan) was used to mix the ingredients. After kneading, the dough was placed in a dish (140 mm × 140 mm × 5 mm), covered with plastic wrap, and allowed to rise for 30 min at 5 °C. The dough was then formed into 5 mm sheets using a rolling pin and then rolled to a thickness of 2 mm using a noodle-making machine (MC200, Nippon Kneader Co., Fujisawa City, Japan). Subsequently, 4 mm-wide fresh noodles were obtained using a noodle-cutting machine. The fresh noodles were hung on a pole and dried at 25 °C for 72 h to produce dried noodles. The doughs prepared using APF, IBF, and OBF were labeled as APF-D, IB-D, and OB-D, while the boiled noodles prepared using these dough types were labeled as APF-N, IB-N, and OB-N. IB or OB was added to APF at three different substitution rates (10%, 30%, or 50%), and the corresponding samples are referred to as IB30%-D or OB50%-N.

### 2.4. Imaging

The noodles and manufacturing process were photographed using a digital camera (WG-40W, Ricoh, Tokyo, Japan). Scanning electron microscopy (SEM) was used to observe the surface structures of the APF, IBF, and OBF. The sample flours were fixed to an SEM specimen stand (Nissin EM Corporation, Type-HM, Ota-ku, Japan) with double-sided carbon tape for SEM (Nissin EM Corporation, 8 mm × 20 m, Tokyo, Japan). Gold deposition was performed, and the specimens were observed via SEM (JSM-IT800SHL, JEOL Ltd., Akishima City, Japan) at an acceleration voltage of 10 kV and a magnification of 150×.

### 2.5. Water Content

The water contents of the dough and dried noodles were measured. Water was evaporated in both samples using a heat drying method (135 °C, 3 h, ordinary pressure), and the water content was calculated using the weight measured before and after the evaporation process.

### 2.6. Color Analysis

The L*, a*, and b* values of the dough and boiled noodles were determined using a spectrophotometer (CR-13, Konica Minolta, Chiyoda-ku, Japan). The dough surface was analyzed after kneading and allowing the dough to rest. The diameter of the measuring lens of the spectrophotometer used was 8 mm; therefore, a boiled noodle sample that completely covered the lens (5 cm × 4 cm) was prepared for color measurements. The dough was rolled, cut into 5 cm × 4 cm pieces, dried at 25 °C for 72 h, and boiled for 10 min to obtain the boiled noodle samples. Ten color measurements were performed for each treatment and the average values were estimated and presented.

### 2.7. Extensibility

For extensibility tests, the prepared doughs were used after they were allowed to rise for 30 min. The dough samples (2 cm in diameter and 10 cm in length) were pulled horizontally at a rate of 5 mm/s. The total length at the time of tearing was measured, and the initial length (10 cm) was subtracted. Four replicates were used for each treatment and the average values are presented.

### 2.8. Gluten Content

Saltwater (500 mL), at the same concentration as that used to prepare the noodles, was added to 20 g of dough from each sample group that had been allowed to rise for 30 min. The dough was washed in saltwater solution 10 times until the water was clear. The surface of the sample was then carefully wiped and weighed. The amount of gluten was calculated from the weight before and after washing with salt water.

### 2.9. Texture Profile

The texture profiles of the dough and boiled noodles were measured. The dough was rolled to a thickness of 2 mm and the noodle strips were cut into samples (diameters = 4.0 cm). For the noodle dough breaking tests, a creep meter (RE2-3305S, Yamaden Corporation, Bunkyo-ku, Japan) was used. The dough sample was placed on a TX-12 sample holder and a 5 mm cylindrical plunger (No. 65, Yamaden Corporation) was used to puncture the dough. Each dough sample was measured 10 times using a 20 N load cell at 1 mm/s measurement speed and 500% strain rate. The breaking strain ratio, total energy, and breaking stress, of each sample were obtained using the breaking strength analysis software program (Yamaden Corporation).

The boiled noodles were prepared by boiling dried noodles for 10 min and cooling them in cold water for 1 min. The texture profiles of the noodles were measured using a creep meter (RE33005, Yamaden Corporation) with a No. 49 wedge-shaped plunger made of acrylic resin (1 mm) using a 20 N load cell at a measurement speed of 1 mm/s and a strain rate of 97%. Because there was a possibility that the noodles may have become soggy during the creep tests, a preliminary experiment was conducted to confirm that there was no change in the physical properties during testing. The breaking strain ratio, total energy, breaking stress, and elastic modulus values of all boiled noodle samples were obtained using the breaking strength analysis software program (Yamaden Corporation).

The breaking strain ratio value indicates the degree of deformation of the sample at the time of fracture. The total energy is the amount of work performed from the first to last measurement point (the entire waveform) and was calculated as the product of the measured stress and strain. The breaking stress is the force per unit area of the breaking load. The elastic modulus is a measure of the resistance of a sample to deformation. The elastic modulus (Pa) was obtained from the slope of the tangent line obtained by regression analysis of the relationship between the stress and strain for the linear range of the curve.

### 2.10. Sensory Evaluation

The dried noodles were boiled for 10 min, cooled in cold water, and used as samples for sensory evaluation. The boiled noodles, together with the noodle soup, were served to the panelists (without sensory training), who were students and faculty members of Shimane University. The panelists consisted of a total of 17 people: 6 males aged 20–29 years, and 11 females aged 19–51 years. The number of sensory evaluation panelists was performed based on previous research findings [16,17,18]. Discrimination tests consisted of color, flavor, hardness, stickiness, and chewiness, whereas preference tests consisted of appearance, flavor, taste, texture, and an overall evaluation, all of which were evaluated using a five-point rating system, ranging from −2 (weakly undesirable characteristics) to +2 (strongly desirable characteristics). The panelists rinsed their mouths thoroughly with water between tasting different samples. Sensory evaluation was conducted with the approval of the Ethical Review Committee for Research Involving Human Subjects at the Faculty of Human Sciences of Shimane University (Approval No.: 2023-11). The data obtained were statistically processed using the method shown in Section 2.13., according to [4]. The panelists signed a prior informed consent form.

### 2.11. Differential Scanning Calorimetry (DSC)

The gelatinization properties of the dough prepared with dry flour were measured using DSC (DSC-60plus, Shimadzu Corporation, Nakagyo-ku, Japan). The calorimeter was calibrated with indium. Approximately 2 mg of the dough sample was weighed into an aluminum DSC pan, and deionized water was added to achieve a water–powder ratio of 2:1. The sealed sample pans were equilibrated at 4 °C for 24 h before analysis and then heated over the temperature range of 25–95 °C at a heating rate of 10 °C/min. An empty sample pan was used as the reference. The ratio of the enthalpy of gelatinization of each sample to that of the control was used to determine the amount of nongelatinized starch [19].

### 2.12. β-Glucan Content, TPC, and Antioxidant Activity

#### 2.12.1. β-Glucan Content

The β-glucan content was quantitatively determined using an enzymatic assay according to the mixed-linkage β-glucan assay procedure (Megazyme Ltd., Bray, Ireland) [20], AOAC method 995.16 [21], AACC method 32–23 [22], and ICC standard method No. 166 [23]. Absorbance was measured at 510 nm using a Microplate Reader (SH-9000Lab, Corona Electric Co., Ltd., Ibaraki, Japan). Each measurement was repeated four times.

#### 2.12.2. TPC

Sample solutions were prepared by mixing 1.0 g of each sample with 10 mL of 60% ethanol and extracting the mixture in an agitating water bath (BW201, Yamato Scientific Co., Ltd., Chuo-ku, Japan) at 40 °C for 2 h at 150 revolutions per minute. The TPC was determined according to a method described in a previous study [24]. Each sample was measured in six replicates and the results were expressed as (+)-catechin equivalents (mg CTN eq/100 g DW).

#### 2.12.3. Antioxidant Activity

Antioxidant activities were assessed using different methods, including hydrophilic-oxygen radical absorbance capacity (H-ORAC), and 2,2-diphenyl-1-picrylhydrazyl (DPPH). The H-ORAC assay was performed according to a previous report [25]. Briefly, the sample solutions were diluted with buffer (75 mM phosphate buffer, pH 7.4) and filtered through a 0.45 µm filter (Advantec Toyo Kaisha, Ltd., Chiyoda-ku, Japan). In a 96-well microplate (#3072, Becton-Dickinson, Franklin Lakes, NJ, USA), fluorescein and AAPH solutions were added to each well containing either the sample solutions or 6-hydroxy-2,5,7,8-tetramethylchroman-2-carboxylic acid (Trolox) solution. The fluorescence intensity (excitation wavelength: 485 nm, emission wavelength: 520 nm) was then measured continuously at 2 min intervals for 90 min using a microplate reader (SH-9000Lab) maintained at 37 °C. Each sample was measured in four replicates and the H-ORAC values were expressed as Trolox equivalents (µmol TE/g dry weight (DW)). DPPH radical scavenging activity was assessed using the modified method proposed by Tsurunaga et al. [26]. Briefly, sample solution (70 μL) and 2-(N-morpholino) ethane sulfonic acid buffer-DPPH (210 µL) were added to a 96-well plate. The mixture was incubated at room temperature for 20 min and the absorbance at 540 nm was measured using a microplate reader (SH-9000Lab). Each sample was measured in six replicates and the DPPH values were expressed as Trolox equivalents (µmol TE/g DW).

### 2.13. Statistical Analysis

All data measurements were statistically analyzed using SPSS (version 28.0, SPSS Inc., Chicago, IL, USA). To determine differences among groups, one-way analysis of variance (ANOVA) was used, followed by Tukey’s test for multiple comparisons. *p*-values < 0.05 were considered statistically significant.

## 3. Results and Discussion

### 3.1. Characteristics of the Raw Materials Used in This Experiment

**Figure 1:**
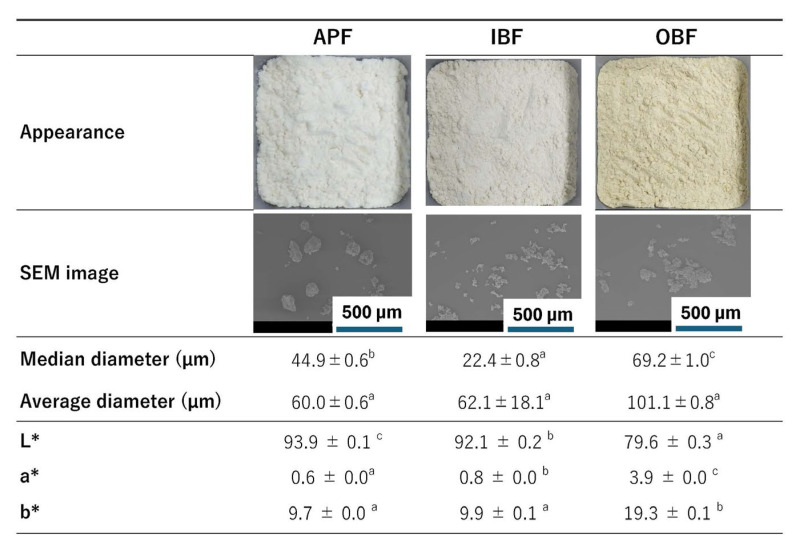
Photographs, scanning electron microscopy (SEM) images, median diameters, average diameters, and lightness (L*), red-green (a*), and yellow-blue (b*) values of the studied flours. APF, all-purpose flour; IBF, inner bran flour; OBF, outer bran flour. Different letters indicate significant differences (*p* < 0.05). Data are expressed as mean ± standard error (SE) (*n* = 4 for median diameter, and average diameter, *n* = 10 for color).

The general compositions, dietary fiber contents, and β-glucan contents of the APF, IBF, and OBF are summarized in Table 1. Compared with APF, IBF and OBF have higher calories, proteins, fats, ash, dietary fiber content, β-glucan content, and TPC. IBF and OBF have lower carbohydrate contents than APF. In general, it is reported that cereal bran contains large amounts of proteins [27], fat [27], ash [28], dietary fibers [29], and polyphenols [27]. In addition, it is known that the edible parts of WB contain considerable amounts of β-glucan. Furuichi et al. [4] reported that IBF and OBF also have high β-glucan contents. The photographs, SEM images, median diameters, average diameters, and color parameters of the flours are shown in Figure 1. The SEM images and particle-size values indicate that APF contains spherical particles with a median diameter of 44.9 µm, IBF particles are irregularly shaped with a median diameter of 22.4 µm (smaller than that of APF), and OBF contains agglomerates of irregularly shaped particles with a median diameter of 69.2 µm. There were no significant differences in the average diameters between APF (60.0 µm), IBF (62.1 µm), and OBF (101.1 µm).

The lightness (L*) values are in the order APF > IBF >> OBF, red-green (a*) values are in the order OBF >> IBF > APF, and the yellow-blue (b*) values follow the order OBF >> IBF ≅ APF (Figure 1). Significant differences are observed in the average L* and a* values of APF and IBF (*p* < 0.05), but the differences are small. OBF exhibits significantly lower L* values and significantly higher a* and b* values than APF and IBF. Therefore, the substitution of APF by IBF will not significantly change the color of the noodles, but the substitution of APF by OBF is expected to darken the color and enhance the red and yellow hues. The similarities between the APF and IBF colors and the stronger yellow hue of OBF are evident from the photographs in Figure 1.

### 3.2. Effect of WB Bran Substitution on the Appearance of Doughs and Noodles

**Figure 2:**
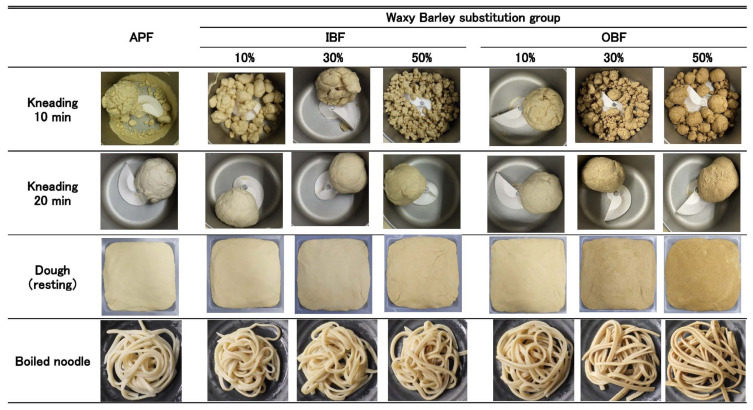
Effects of IBF or OBF substitution on the formations of the doughs and boiled noodles.

The photographs of the manufacturing process, dough, and boiled noodles are shown in Figure 2. After 10 min of kneading, some mixtures did not produce a cohesive dough; however, after 20 min of kneading, all mixtures produced a cohesive dough. The substitution of a part of APF with IBF or OBF did not reduce the workability of the dough during the manufacturing process, such as rolling and cutting steps. Figure 2 shows the appearance of the dough and boiled noodles prepared from APF with different amounts of IBF or OBF. The OB30%-D and OB50%-D and OB30%-N and OB50%-N samples have a darker hue.

**Figure 3:**
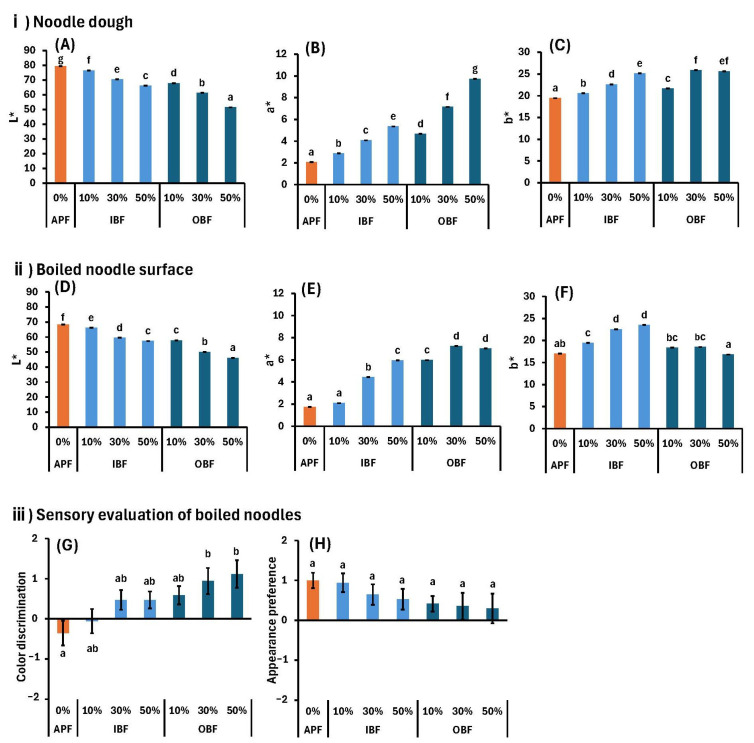
Effects of APF substitution with IBF or OBF on the (**A**–**C**) color of the doughs, (**D**–**F**) boiled noodles, and (**G**,**H**) sensory evaluation results for the boiled noodles. Different letters indicate significant differences at *p* < 0.05. Data are expressed as mean ± SE (*n* = 10 for color, *n* = 17 for sensory evaluations).

Figure 3A–C shows the color results for the doughs, and Figure 3D–F shows similar results for the boiled noodle surfaces. Compared with those of APF, the L* values were lower for both the dough (Figure 3A) and boiled noodles (Figure 3D) after IBF or OBF substitution, and decreased at increasing substitution rates (Figure 3A,D). Comparing the groups with the same substitution ratio of IBF and OBF, OBF substitution resulted in significantly lower L* values for both the doughs and boiled noodles than IBF substitution (Figure 3A,D).

The a* values were higher for the IBF- and OBF-substituted samples of both doughs than those for the APF dough (Figure 3B) and boiled noodles (Figure 3E), with the values increasing as a function of the substitution rate. This trend was more pronounced in the dough than in the boiled noodles (Figure 3B,E). At the same substitution rates, the a* values were higher for the OB dough and boiled noodles than those for the IB samples (Figure 3B,E).

The b* value tended to increase with IBF substitution compared to APF substitution, in both doughs (Figure 3C) and boiled noodles (Figure 3F). However, the b* value of the OB-D was higher than that of APF-D (Figure 3C), but the boiled noodles showed similar values at all substitution rates (Figure 3F).

The L* values of raw APF, IBF, and OBF follow the order APF > IBF >> OBF, and the a* values follow the order: OBF >> IBF > APF (Table 1). Thus, it is considered that the L* and a* values of the dough and boiled noodles reflect the color properties of the raw materials. The b* values of the raw flours follow the order OBF >> IBF = APF; by contrast, the order for the boiled noodles is different, i.e., IB-N > OB-N = APF-N. The a* values of the doughs and boiled noodles at 30% and 50% OB substitution rates are high (Figure 3B,E), giving a darker appearance (Figure 2). This is because the yellow color is weak, and the b* value of the raw material is not easily reflected. Therefore, the L* and a* values are more likely to follow the color of the raw material.

The sensory evaluation results for color discrimination and appearance preference for the boiled noodles are shown in Figure 3G,H. Color discrimination was scored on a scale of −2 (weak) to +2 (strong); the scores for OB30%-N and OB50%-N are 0.94 and 1.1, respectively, indicating that the color was perceived to be much darker than those of the other groups (Figure 3G). For visualization purposes, scores were allocated on a scale of −2 (unfavorable) to +2 (favorable) on five levels. However, unlike color discrimination evaluation, no significant differences were found in the appearance preference (Figure 3H). Although the measured L*, a*, and b* values of the boiled noodles changed considerably due to OBF substitution (Figure 3D–F), the sensory evaluation results showed that the appearance of these noodles was preferred; no significant differences were recorded among the panelists for these types of noodles compared with those prepared using only APF. In addition, among the L*, a*, and b* values, the a* values show a trend that is most similar to the color discrimination sensory evaluation results (Figure 3E), suggesting that when the noodles are reddish, people perceive them as strong in color. These results show that the colors of the dough and boiled noodles are affected considerably by IBF or OBF substitution through objective color analysis. However, the preferences of human panelists are less sensitive to color differences.

### 3.3. Effects of WB Bran Substitution on Water Content, Dough Extensibility, Gluten Content, and Thermal Properties of Dough

Table 2 shows the water content of the dough and dried noodles. The water content of the dough was in the range of 36.66–38.58%, while that of the dried noodles was in the range of 9.5–10.67%. It is considered that the water content of dried noodles should be ≤15%, and previous reports have also shown that it is 11.74–12.5% [30,31], so it was found that the noodle samples were sufficiently dried under the drying conditions (25 °C, 72 h) of this study.

**Figure 4:**
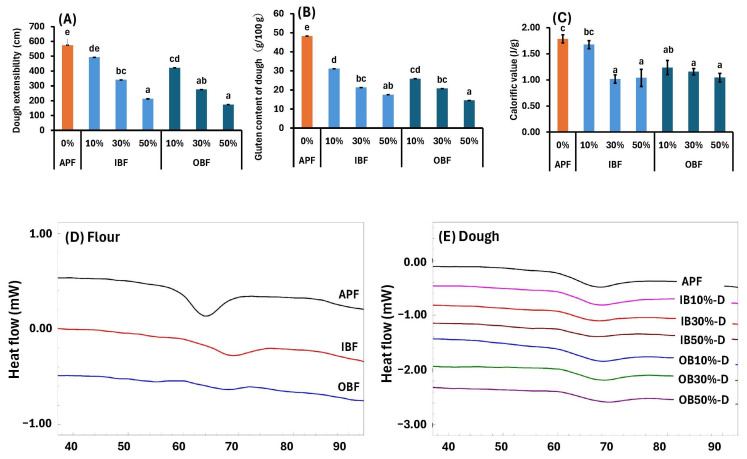
Effects of IBF or OBF substitution on the (**A**) extensibility, (**B**) gluten content, (**C**) calorific value of the doughs, (**D**) DSC curves of flour, and (**E**) DSC curves of dough. Different letters indicate significant differences at *p* < 0.05. Data are expressed as mean ± SE (*n* = 4).

The chewy texture of noodles is affected by the gluten network formed during manufacturing [32]. Although barley contains hordein, which has a function similar to that of wheat gluten [33], IBF and OBF used in this study are bran parts (Figure 1). Therefore, it is predicted that the gluten network formation of the noodles would be inhibited [34], and the chewy texture of the noodles would be weakened. Therefore, to understand the effects of IBF and OBF substitution on gluten formation, we measured the extensibility and wet gluten contents of the dough (Figure 4).

Both the extensibility and wet gluten content were the highest in APF-D, and lower in IB-D and OB-D. Furthermore, as the IBF and OBF substitution rates increased, the extensibility and wet gluten content decreased. At the same substitution rate, no differences were observed between IB-D and OB-D. Polyphenols interact with gluten proteins in complex ways, they can reportedly inhibit or enhance the function of gluten. When tea polyphenols are added to bread dough, the volume of bread decreases, and the elasticity of the dough increases, which can lead to a decrease in bread quality. This is reportedly due to a decrease in disulfide bond strength and changes in the secondary structure of the protein [35]. In contrast, some studies have found that tea polyphenols promote the formation of the gluten network, strengthen the hydrophobic interactions and α-helix structure of gluten proteins, and improve the texture of noodles by increasing the stability and chewiness of the noodle dough [36]. Similarly, barley bran is rich in polyphenols [37], and contains diosmetin, luteolin, protocatechuic acid, vanillic acid, ferulic acid, phlorogucinol, and diosmin [38]. In particular, WB bran contains a large amount of proanthocyanidins [39]. It has been shown that proanthocyanidins inhibit gluten formation by preferentially binding to and cross-linking the polymeric glutelin subunits via hydrophobic interactions and hydrogen bonds [40]. Considering this background, it is suggested that the APF substitution with IBF and OBF inhibits gluten formation; however, this is due to the decrease in the amount of wheat gluten and the effects of the proanthocyanidins in WB.

Furthermore, the thermal properties of dried dough were investigated using DSC [19]. The calorific value of APF-D is 1.79 J/g (Figure 4C), and the values for all the sample groups except IB10%-D were significantly lower (*p* < 0.05), indicating that the energy required for the gelatinization of starch during heating was reduced upon bran addition. In the DSC curves for the flour ingredients, a distinct peak was observed at approximately 64 °C for APF, but the peaks were much smaller for IBF and OBF (Figure 4D). In the dried dough case, a distinct peak was observed for APF, but the peaks were small for IB-D and OB-D dried noodles, except those of IB10%-D (Figure 4E). This is presumably due to the low-starch contents of IBF and OBF and the influence of lipids and ash. Compared with APF, IBF and OBF have higher fat and ash contents (Figure 1). Because fatty acids [41] and ash [42] reportedly alter gelatinization properties, the increase in fatty acid and ash contents due to bran addition is believed to decrease the enthalpy measured by DSC.

From these results, it became clear that when a part of the APF was replaced with WB, the extensibility, gluten content, and thermal properties of the dough decreased significantly. This is because of the effects of proanthocyanidins, fatty acids, and ash contained in WB.

### 3.4. Effects of WB Bran Substitution on Physical Properties and Sensory Evaluation of Dough and Boiled Noodles

**Figure 5:**
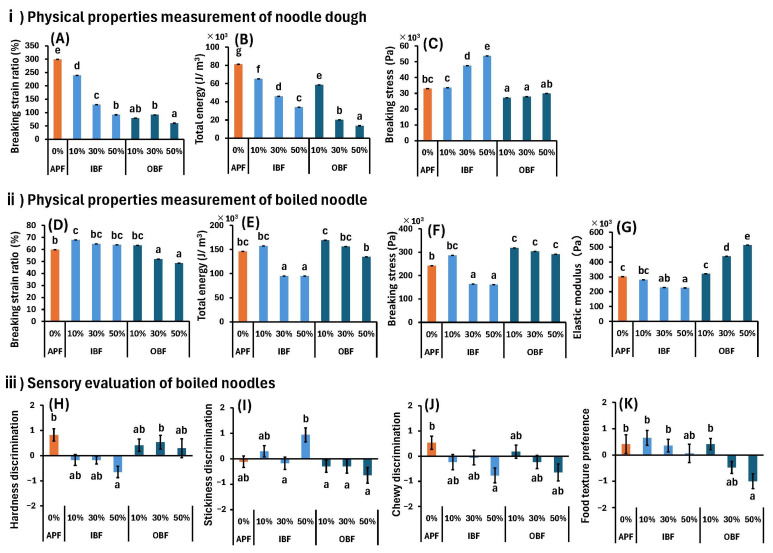
Effects of APF substitution with IBF or OBF on the physical properties of the (**A**–**C**) doughs, (**D**–**G**) boiled noodles, and (**H**–**K**) sensory evaluation results for the boiled noodles. Different letters indicate significant differences at *p* < 0.05. Data are expressed as mean ± SE (*n* = 10 for physical properties measurements, *n* = 17 for sensory evaluations).

The results of the physical and sensory evaluations of the dough and boiled noodles are shown in Figure 5. The breaking strain ratio, total energy, and breaking stress values of the dough were measured using penetration tests (Figure 5A–C). The breaking strain ratios and total energies of IB-D and OB-D were lower than those of APF-D (Figure 5A,B), indicating that the degree of deformation (elongation) and energy required for dough tears are lower for IB-D and OB-D. Furthermore, when comparing IB-D and OB-D at the same substitution rate, OB-D exhibits a lower breaking strain rate and total energy (Figure 5A,B). The breaking stress, which indicates the stress required for food to break, was significantly higher in the IB30%-N and IB50%-N group cases compared with that in the APF group case (Figure 5C), indicating that these strong doughs are difficult to tear. In contrast, OB10%-D and OB30%-D exhibited lower breaking stresses than APF and were observed to tear easily (Figure 5C). In summary, although the degree of deformation of the dough and the energy required for tears are low for IB-D, the stress required for tears is high. For OB-D, the degree of deformation, energy required for rupture, and the stress required for tear are smaller than those for APF-D. As mentioned in Section 2.3, the breaking characteristics of the dough would be expected to change because the extensibility and gluten content of the dough would decrease if APF was replaced with IBF or OBF (Figure 4).

For boiled noodles, the breaking strain ratio (Figure 5D), total energy (Figure 5E), breaking stress (Figure 5F), and elastic modulus (Figure 5G) were analyzed when the noodles were cut with a plunger (width = 1 mm). The breaking strain ratio was significantly higher for IB10%-N than for APF-N and significantly lower for OB30%-N and OB50%-N than for APF-N (Figure 5D). The total energies of IB30%-N and IB50%-N were significantly lower IB than that of APF-N (Figure 5E). The breaking stress was significantly lower for IB30%-N and IB50%-N than APF-N and significantly higher for all OB-N samples than APF-N (Figure 5F). The elastic modulus values of IB30%-N and IB50%-N were significantly lower than that of APF-N, while the values for OB30%-N and OB50%-N were significantly higher (Figure 5G). These results confirm that the total energy, breaking stress, and elasticity modulus values of IB30%-N and IB50%-N are significantly lower than those of APF-N, indicating that they can tear more easily with a small amount of force and have lower elasticity. In contrast, OB30%-N and OB50%-N have significantly lower breaking strains and significantly higher breaking stresses and elastic moduli than APF-N. This indicates that although the chewy texture of the noodles made from OB30%-N and OB50%-N is weaker than that of APF-N, they are harder to break.

When comparing the results for the doughs (Figure 5A–C) and boiled noodles (Figure 5D–G), the effects of IBF or OBF substitution on all physical properties are remarkably different. Compared with the texture of APF-D, the chewy textures of IB30%-D and IB50%-D are weaker (Figure 5A) and these doughs are more difficult to break (Figure 5C); however, there are no significant differences in the chewy textures of IB30%-N and IB50%-N with respect to that of APF-N (Figure 5D). Nevertheless, these noodles are easier to break (Figure 5F). All OB-D samples have weaker chewy textures than that of APF-D (Figure 5A) and are more likely to break (Figure 5C); however, the boiled noodles are less likely to break and have a significantly stronger chewy texture (Figure 5F,G). The physical properties of the doughs (Figure 5A–C) and boiled noodles (Figure 5D–G) are different owing to the various structural changes in the macromolecular substances caused by boiling the noodles.

For example, heating causes the three-dimensional protein structure to relax, making it easier for proanthocyanidins in IBF and OBF to bind with the protein through hydrophobic interactions and hydrogen bonds [40] to form complexes that can change the physical properties. The outer layer of cereal bran reportedly contains more proanthocyanidins than the inner layer [43]; thus, the formation of protein–proanthocyanidin complexes is believed to be one of the reasons for the higher total energy, breaking stress, and elastic modulus of OBF than those of IBF at the same substitution rates (Figure 5E–G). However, there are some inconsistencies associated with this hypothesis. If the increases in the total energy, breaking stress, and elastic modulus of boiled noodles due to OBF substitution are solely due to the proanthocyanidin content, these values should increase as the OBF substitution rate increases. However, no correlation was observed between the substitution rate and the total energy and breaking stress (Figure 5E,F). We believe that this lack of correlation is due to the counterbalancing of the strengthening effect of the protein–proanthocyanidin complexes by the weakening effect induced by the reduction in wheat protein content due to IBF and OBF substitution. In addition, the cooking process can cause the partial solubilization of β-glucan, increasing the viscosity of the noodle and making it more difficult to break. Izydorczyk et al. prepared fresh yellow alkaline noodles by substituting some wheat with barley, which is rich in β-glucan, and reported that the overall hardness of the noodles increased considerably [44]. They stated that the viscoelasticity and network-forming properties of the ingredients contained in barley resulted in the formation of a semisolid network that contributed to noodle hardness [44]. The β-glucan contents of IBF and OBF were 4.8 and 3.9 g/100 g, respectively (Table 1), with IBF having the highest content. The breaking strain ratio, which is related to the firmness of noodles, was higher for IB-N than for APF-N or OB-N, which could be due to the high β-glucan content of IB-N. These results show that the breaking strain ratio and total energy of the dough decrease significantly owing to the substitutions of APF by IBF and OBF. However, the formation of the protein–proanthocyanidin complexes and the solubilization of β-glucan due to the weakening of the protein structure during heating occur in the boiled noodles, thereby strengthening their textures. Although the substitution of IBF and OBF is negative for the noodle dough, the texture changes when it is boiled. It was shown that it is possible to prepare noodles with a texture equivalent to or better than APF-N by adjusting the amount of replacement of IB-N and OBF-N.

The results of the sensory evaluation of the boiled noodle textures are shown in Figure 5H–K. In the discriminant evaluation conducted to determine the strength of each parameter, the hardness (Figure 5H), stickiness (Figure 5I), and chewy textures (Figure 5J) of the boiled noodles were scored from −2 (weak) to +2 (strong). The results showed that the IB50%-N has lower hardness (Figure 5H) and chewy texture (Figure 5J) compared with the equivalent characteristics of APF-N, but no significant stickiness difference was observed compared with that of the APF-N (Figure 5I). In the texture preference test, the textures were scored from −2 (unfavorable) to +2 (favorable), with only OB50%-N scoring unfavorably compared with APF-N (Figure 5K). The results of the measurements conducted using the aforementioned physical property measurement device (Figure 5D–G) showed that there was a significant impact from the substitution of IBF and OBF, and the only differences between APF-N and the other samples in sensory evaluations were the hardness and chewy textures of IB50%-N in the discriminant evaluation and the preference of OB50%-N in the preference test (Figure 5H,J,K). Based on these results, it is concluded that the physical property measurement device used in these experiments has a higher sensitivity to texture than humans. The breaking strain ratio results (Figure 5D) yielded the highest similarities with the preference results (Figure 5K), suggesting that this parameter could be used to predict noodle preferences.

### 3.5. Effects of WB Bran Replacement on Aroma, Taste, and Overall Impression in Sensory Evaluations

**Figure 6:**
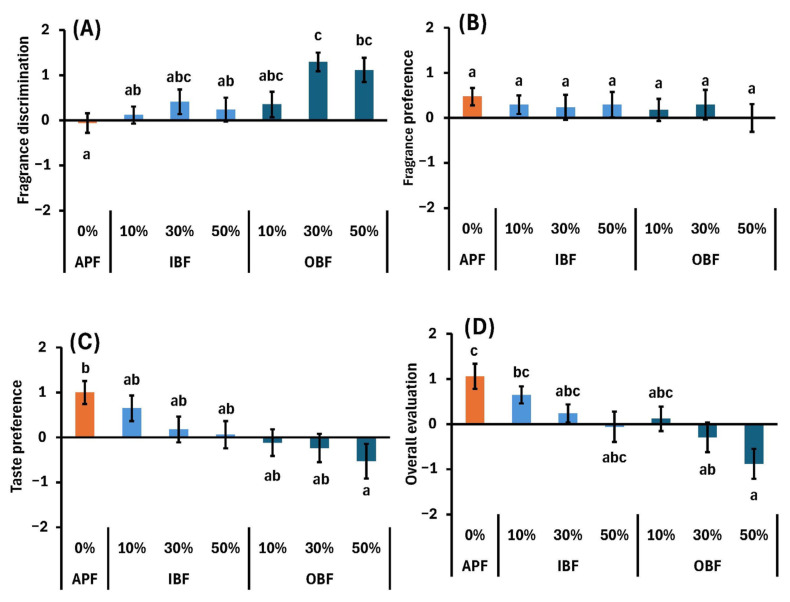
Effects of IBF or OBF substitutions on the sensory parameters of boiled noodles: (**A**) fragrance discrimination, (**B**) fragrance preference, (**C**) taste preference, and (**D**) overall evaluation. Different letters indicate significant differences at *p* < 0.05. Data are expressed as mean ± SE (*n* = 17).

In addition to the appearance and texture sensory parameters shown in Section 2.2 and Section 2.4, respectively, other sensory parameters are shown in Figure 6. Compared with APF-N, OB30%-N and OB50%-N exhibited strong aromas in the fragrance discrimination tests (Figure 6A). Gas chromatography–mass spectrometry results showed that IB and OB contained large amounts of acetic acid and 2,3-butanediol [4]. Acetic acid is a type of carboxylic acid found in vinegar; it has a strong pungent odor and has been reported as an aroma component of cooked barley [45]. Furthermore, 2,3-butanediol is a volatile component of cocoa beans [46] and black rice from Thailand [47], which imparts a sweet aroma. However, as there were no differences in the evaluations of aroma preferences among the sample groups, it can be inferred that the strong aromas of OB30%-N and OB50%-N (Figure 6A) were not sufficient to influence consumer preference (Figure 6B). In terms of taste preference, OB50%-N has a lower score (−0.53) than that of APF-N (1.0) (Figure 6C). Although we did not analyze the taste components of OB-N, it is known that barley bran contains many off-flavors such as bitterness [48]. This off-flavor is thought to be the cause of the lower taste preference ratings. The results of the overall evaluation that takes into account all preference parameters are shown in Figure 6D. The overall preference scores were significantly lower for OB30%-N (−0.3) and OB50%-N (−0.9) compared with those for APF-N (1.1) (Figure 6D), which was thought to be attributed to their low ratings for texture (Figure 5K) and taste preferences (Figure 6C).

### 3.6. Effects of WB Bran Substitution on β-Glucan Content and Antioxidant Properties

**Figure 7:**
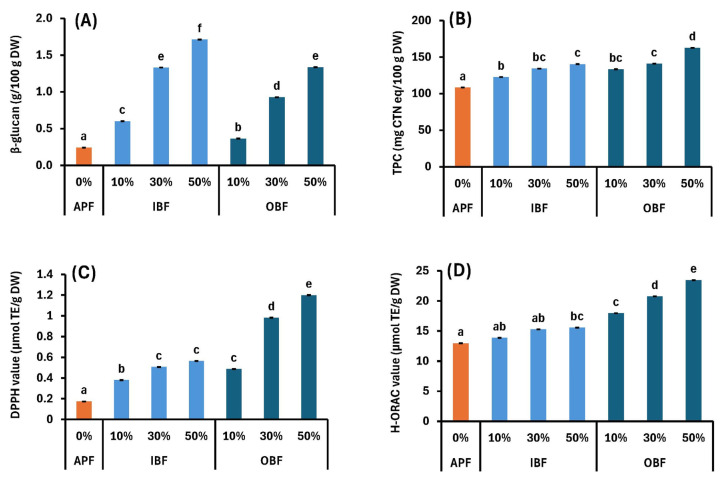
Effects of IB or OB substitution on (**A**) β-glucan content, (**B**) total polyphenol content (TPC), (**C**) 2,2-diphenyl-1-picrylhydrazyl (DPPH) value, and (**D**) hydrophilic oxygen radical absorbance capacity (H-ORAC) values of dried dough. Different letters indicate significant differences at *p* < 0.05. Data are expressed as mean ± SE (*n* = 4 for β-glucan, *n* = 6 for TPC, and DPPH, *n* = 4 for H-ORAC).

The β-glucan contents and antioxidant properties of the dried dough are presented in Figure 7A–D. The β-glucan content of the dried dough increases significantly as the IBF and OBF substitution rate increases. In particular, as the β-glucan content of IBF is higher than that of OBF (Table 1), IBF has significantly higher values at the same substitution rate.

β-glucan has been reported to induce significant reductions in the levels of total cholesterol and low-density lipoprotein cholesterol, which are risk factors for cardiovascular disease [49]. It is also useful for managing diabetes by improving blood glucose control during fasting and after meals [50]. Furthermore, it has been reported to strengthen the immune system [51], improve intestinal flora, provide anti-inflammatory effects, and reduce oxidative stress [51,52]. In addition, the TPC, DPPH, and H-ORAC values tend to increase significantly as the IB and OB substitution rates increase (Figure 7B–D). OBF has a higher TPC and stronger antioxidant properties than IBF [4]. The TPC and antioxidant properties of the IB-N and OB-N groups are enhanced because of the antioxidant properties of bran. It has been reported that barley bran contains polyphenols, such as diosmetin, luteolin, protocatechuic acid, vanillic acid, ferulic acid, phlorogucinol, and diosmin [38]; in particular, WB bran contains a large number of proanthocyanidins [39]. Many studies have investigated the antioxidant properties and health benefits of these components, including the prevention of cardiovascular disease [53] and cancer [54], anti-aging effects [55], and strengthening of the immune system [56].

Therefore, β-glucan and antioxidants have many health benefits, and noodles with enhanced quantities of these components are expected to contribute to maintaining good health.

In this study, we investigated the use of IBF and OBF to enhance noodle health benefits and assess their impact on quality. WB bran substitution increased β-glucan and antioxidant contents but affected color, texture, and sensory properties. IB substitution up to 50% maintained acceptable quality and palatability, while OB substitution above 10% reduced taste and texture preferences. The findings presented herein demonstrated the feasibility of repurposing WB bran (a food industry by-product) for nutrient-enriched noodles, superior sensitivity of analytical testing compared with sensory evaluations, and substitution thresholds that balance health functionality and quality. However, there are some issues associated with this study. The sensory evaluation in this study was conducted by panelists who had not received any sensory testing training. For this reason, it is impossible to dismiss the possibility that the sensory evaluation results were not as sensitive as those obtained using instrumental analyses.

## 4. Conclusions

In this study, noodles were produced by partially substituting APF with IBF or OBF. The substitution of a part of the wheat flour with IBF or OBF had a significant effect on the appearance, color, physical properties, and sensory characteristics of the dough and boiled noodles. The analytically measured color and physical properties showed a clear correlation with the IB or OB substitution rate; however, the differences between the sample groups were small in the sensory evaluation results. In terms of the analytically measured physical properties, the substitution of IBF and OBF resulted in lower values of texture parameters for dough, but the texture of boiled noodles was improved considerably. In addition, the β-glucan and antioxidant contents and TPC increased significantly as the IB or OB substitution rate increased. Therefore, the addition of IBF or OBF was found to improve effectively the health functionality of wheat noodles. However, sensory evaluation results showed no detectable changes in quality and taste preferences compared with APF up to 50% IBF substitution, while taste preference and overall evaluation decreased significantly at OBF substitutions of 30% and 50%. Therefore, considering quality, palatability, and health functionality, APF can be substituted with IB at a rate of 50%, while the substitution of OBF is limited to values ≤10%. Regarding physical properties, the texture of dough made with IB-N or OB-N was lower in strength than that made with APF-N, but the texture of boiled noodles made with IB-N or OB-N was better, and this was particularly noticeable in the elastic modulus. These findings clarified the WB bran substitution rates that can improve health functionality of wheat noodles without reducing quality.

In this study, we examined noodles, where the formation of wheat gluten has a significant impact on quality, but we would also like to consider its use in Western confectionery products, such as cookies, which do not require the formation of gluten. In the case of wheat flour products that do not use gluten formation, even if the amount of IBF and OBF increases, unlike noodles, it is thought that there will be almost no effect on the product.

WB bran is currently disposed of as waste in the food industry. If it were possible to manufacture high-quality, high-function foods that make use of the functionality of barley bran, a by-product of food waste, this would, thus, contribute to the realization of a recycling-oriented and sustainable growth society that supports global food supplies. We hope to conduct additional in-depth research studies and develop foods that make effective use of WB bran.

## Figures and Tables

**Table 1:** General composition and dietary fiber, β-glucan, and polyphenol contents of the all-purpose flour (APF), inner bran flour (IBF), and outer bran flour (OBF).

Component	Unit	APF	IBF	OBF
Energy ^1^	kcal/100 g	350	370	402
Protein ^1^	g/100 g	10.9	11.4	14.9
Fat ^1^	g/100 g	1.6	4.5	9.5
Carbohydrates ^1^	g/100 g	74.3	71.0	64.1
Dietary fiber ^1^	g/100 g	2.7	9.1	20.6
Ash ^1^	g/100 g	0.5	2.6	4.1
β-glucan	g/100 g	0.3 ^a^	4.8 ^c^	3.9 ^b^

^1^ The values were provided in a commissioned analysis by Bureau Veritas FEAC. β-glucan content was determined from our analysis, and different letters indicate significant differences at *p* < 0.05. Data are expressed as mean ± SE (*n* = 8 for β-glucan).

**Table 2:** Effects of WB bran substitution on water content of dough and dried noodles.

Sample		APF	IBF			OBF		
			10%	30%	50%	10%	30%	50%
Dough	%	36.66 ± 0.42 ^a^	38.57 ± 0.41 ^c^	37.66 ± 0.10 ^abc^	37.09 ± 0.07 ^ab^	38.28 ± 0.29 ^bc^	37.58 ± 0.09 ^abc^	37.33 ± 0.10 ^ab^
Dried Noodle	%	10.40 ± 0.02 ^c^	10.67 ± 0.05 ^d^	9.93 ± 0.07 ^b^	10.09 ± 0.06 ^b^	11.77 ± 0.06 ^e^	9.90 ± 0.04 ^b^	9.50 ± 0.01 ^a^

Different letters indicate significant differences at *p* < 0.05. Data are expressed as mean ± SE (*n* = 3).

## Data Availability

The original contributions presented in this study are included in the article. Further inquiries can be directed to the corresponding author.

## References

[1] Yang J.-L., Kim Y.-H., Lee H.-S., Lee M.-S., Moon Y.K. (2003). Barley β-glucan lowers serum cholesterol based on the up-regulation of cholesterol 7α-hydroxylase activity and mRNA abundance in cholesterol-fed rats. J. Nutr. Sci. Vitaminol..

[2] Messia M.C., De Arcangelis E., Candigliota T., Trivisonno M.C., Marconi E. (2020). Production of ß-glucan enriched flour from waxy barley. J. Cereal Sci..

[3] Ullrich S., Clancy J., Eslick R., Lance R. (1986). β-Glucan content and viscosity of extracts from waxy barley. J. Cereal Sci..

[4] Furuichi T., Abe D., Uchikawa T., Nagasaki T., Kanou M., Kasuga J., Matsumoto S., Tsurunaga Y. (2023). Comparison of Nutritional Composition and Antioxidant Properties of Pulverized and Unutilized Portions of Waxy Barley. Foods.

[5] Hughes J., Grafenauer S. (2021). Oat and barley in the food supply and use of beta glucan health claims. Nutrients.

[6] Tonooka T. (2023). Breeding of Waxy Barley Cultivars in the National Barley Breeding Program of Japan. Jpn. Agric. Res. Q. JARQ.

[7] Harding S.V., Storsley J., Thandapilly S.J., Ames N.P. (2013). Lower 30 Minute Serum Insulin in Healthy Sprague-Dawley Rats Consuming Chips from Specific Barley Flour Blends. Cereal Chem..

[8] Lupton J.R., Robinson M.C., Morin J.L. (1994). Cholesterol-lowering effect of barley bran flour and oil. J. Am. Diet. Assoc..

[9] Tamagawa K., Iizuka S., Fukushima S., Endo Y., Komiyama Y. (1997). Antioxidative activity of polyphenol extracts from barley bran. J. Jpn. Soc. Food Sci. Technol..

[10] Chen J., Fei M., Shi C., Tian J., Sun C., Zhang H., Ma Z., Dong H. (2011). Effect of particle size and addition level of wheat bran on quality of dry white Chinese noodles. J. Cereal Sci..

[11] Fan L., Ma S., Wang X., Zheng X. (2016). Improvement of Chinese noodle quality by supplementation with arabinoxylans from wheat bran. Int. J. Food Sci. Technol..

[12] Gull A., Prasad K., Kumar P. (2018). Nutritional, antioxidant, microstructural and pasting properties of functional pasta. J. Saudi Soc. Agric. Sci..

[13] Susanna S., Prabhasankar P. (2013). A study on development of Gluten free pasta and its biochemical and immunological validation. LWT-Food Sci. Technol..

[14] Shen S., Chi C., Zhang Y., Li L., Chen L., Li X. (2021). New insights into how starch structure synergistically affects the starch digestibility, texture, and flavor quality of rice noodles. Int. J. Biol. Macromol..

[15] Taniguchi A., Miura M., Ikeda T.M., Kaneko S., Kobayashi R. (2022). Factors affecting rheological properties of barley flour-derived batter and dough examined from particle properties. Food Hydrocoll..

[16] Pu H., Wei J., Wang L., Huang J., Chen X., Luo C., Liu S., Zhang H. (2017). Effects of potato/wheat flours ratio on mixing properties of dough and quality of noodles. J. Cereal Sci..

[17] Cai J., Chiang J., Tan M., Saw L., Xu Y., Ngan-Loong M. (2016). Physicochemical properties of hydrothermally treated glutinous rice flour and xanthan gum mixture and its application in gluten-free noodles. J. Food Eng..

[18] Udachan I., Sahoo A. (2017). Quality evaluation of gluten free protein rich broken rice pasta. J. Food Meas. Charact..

[19] Li M., Zhu K.-X., Sun Q.-J., Amza T., Guo X.-N., Zhou H.-M. (2016). Quality characteristics, structural changes, and storage stability of semi-dried noodles induced by moderate dehydration: Understanding the quality changes in semi-dried noodles. Food Chem..

[20] β-Glucan Assay Kit (Mixed Linkage) Assay Protocol. https://prod-docs.megazyme.com/documents/Assay_Protocol/K-BGLU_DATA.pdf.

[21] AOAC International (2023). AOAC Official Method 995.16;β-D-Glucan in Oats: Streamlined Enzymatic Method.

[22] Cereals & Grains Association (2010). AACC Approved Method 32–23.01; beta-Glucan Content of Barley and Oats—Rapid Enzymatic Procedure.

[23] International Association for Cereal Sciences and Technology (1998). ICC Standard Method No.166; Determination of ß-Glucan in Barley, Oat and Rye.

[24] Ainsworth E.A., Gillespie K.M. (2007). Estimation of total phenolic content and other oxidation substrates in plant tissues using Folin–Ciocalteu reagent. Nat. Protoc..

[25] Watanabe J., Oki T., Takebayashi J., Yamasaki K., Takano-Ishikawa Y., Hino A., Yasui A. (2012). Method validation by interlaboratory studies of improved hydrophilic oxygen radical absorbance capacity methods for the determination of antioxidant capacities of antioxidant solutions and food extracts. Anal. Sci..

[26] Tsurunaga Y., Kanou M., Ikeura H., Makino M., Oowatari Y., Tsuchiya I. (2022). Effect of different tea manufacturing methods on the antioxidant activity, functional components, and aroma compounds of *Ocimum gratissimum*. LWT.

[27] Cherno N., Gural L., Naidonov O. (2020). Black wheat bran as a promising source of food fibres with an expanded spectrum of Functionalities. Grain Prod. Mix. Fodder’s.

[28] Ker Y.-B., Wu H.-L., Chen K.-C., Peng R.Y. (2022). Nutrient composition of *Chenopodium formosanum* Koidz. bran: Fractionation and bioactivity of its soluble active polysaccharides. PeerJ.

[29] Bach Knudsen K.E., Nørskov N.P., Bolvig A.K., Hedemann M.S., Laerke H.N. (2017). Dietary fibers and associated phytochemicals in cereals. Mol. Nutr. Food Res..

[30] Li Y., Wang X., Jiang P., Li X. (2016). Sorption equilibrium moisture and isosteric heat of adsorption of Chinese dried wheat noodles. J. Stored Prod. Res..

[31] Markovic P.J., Stojiljkovic M.J. (2019). The Kinetics of Drying Pasta with Added of Soya Flour. Int. J. Latest Eng. Sci..

[32] Zhang M., Ma M., Yang T., Li M., Sun Q. (2022). Dynamic distribution and transition of gluten proteins during noodle processing. Food Hydrocoll..

[33] Biesiekierski J.R. (2017). What is gluten?. J. Gastroenterol. Hepatol..

[34] Boita E.R., Oro T., Bressiani J., Santetti G.S., Bertolin T.E., Gutkoski L.C. (2016). Rheological properties of wheat flour dough and pan bread with wheat bran. J. Cereal Sci..

[35] Qin W., Pi J., Zhang G. (2022). The interaction between tea polyphenols and wheat gluten in dough formation and bread making. Food Funct..

[36] Han C.-W., Ma M., Zhang H.-H., Li M., Sun Q.-J. (2020). Progressive study of the effect of superfine green tea, soluble tea, and tea polyphenols on the physico-chemical and structural properties of wheat gluten in noodle system. Food Chem..

[37] Gangopadhyay N., Harrison S.M., Brunton N.P., Hidalgo-Ruiz J.L., Gallagher E., Rai D.K. (2018). Brans of the roller-milled barley fractions rich in polyphenols and health-promoting lipophilic molecules. J. Cereal Sci..

[38] Zhang W., Lan Y., Dang B., Zhang J., Zheng W., Du Y., Yang X., Li Z. (2023). Polyphenol profile and in vitro antioxidant and enzyme inhibitory activities of different solvent extracts of highland barley bran. Molecules.

[39] Verardo V., Cevoli C., Pasini F., Gómez-Caravaca A.M., Marconi E., Fabbri A., Caboni M.F. (2015). Analysis of oligomer proanthocyanidins in different barley genotypes using high-performance liquid chromatography–fluorescence detection–mass spectrometry and near-infrared methodologies. J. Agric. Food Chem..

[40] Girard A.L., Bean S.R., Tilley M., Adrianos S.L., Awika J.M. (2018). Interaction mechanisms of condensed tannins (proanthocyanidins) with wheat gluten proteins. Food Chem..

[41] Zhou Z., Robards K., Helliwell S., Blanchard C. (2007). Effect of the addition of fatty acids on rice starch properties. Food Res. Int..

[42] Santiago-Ramos D., de Dios Figueroa-Cárdenas J., Véles-Medina J.J., Salazar R. (2018). Physicochemical properties of nixtamalized black bean (*Phaseolus vulgaris* L.) flours. Food Chem..

[43] Huang Y.-P., Lai H.-M. (2016). Bioactive compounds and antioxidative activity of colored rice bran. J. Food. Drug. Anal..

[44] Izydorczyk M., Lagasse S., Hatcher D., Dexter J., Rossnagel B. (2005). The enrichment of Asian noodles with fiber-rich fractions derived from roller milling of hull-less barley. J. Sci. Food Agric..

[45] Takemitsu H., Amako M., Sako Y., Kita K., Ozeki T., Inui H., Kitamura S. (2019). Reducing the undesirable odor of barley by cooking with superheated steam. J. Food Sci. Technol..

[46] Rodriguez-Campos J., Escalona-Buendía H., Orozco-Avila I., Lugo-Cervantes E., Jaramillo-Flores M.E. (2011). Dynamics of volatile and non-volatile compounds in cocoa (*Theobroma cacao* L.) during fermentation and drying processes using principal components analysis. Food Res. Int..

[47] Ajarayasiri J., Chaiseri S. (2008). Comparative study on aroma-active compounds in Thai, black and white glutinous rice varieties. Agric. Nat. Resour..

[48] Heiniö R.-L., Noort M., Katina K., Alam S.A., Sozer N., De Kock H.L., Hersleth M., Poutanen K. (2016). Sensory characteristics of wholegrain and bran-rich cereal foods—A review. Trends Food Sci Technol..

[49] Xu D., Liu H., Yang C., Xia H., Pan D., Yang X., Yang L., Wang S., Sun G. (2021). Effects of different delivering matrices of β-glucan on lipids in mildly hypercholesterolaemic individuals: A meta-analysis of randomised controlled trials. Br. J. Nutr..

[50] Bozbulut R., Sanlier N. (2019). Promising effects of β-glucans on glyceamic control in diabetes. Trends Food Sci. Technol..

[51] Bai J., Ren Y., Li Y., Fan M., Qian H., Wang L., Wu G., Zhang H., Qi X., Xu M. (2019). Physiological functionalities and mechanisms of β-glucans. Trends Food Sci. Technol..

[52] Chen H., Nie Q., Xie M., Yao H., Zhang K., Yin J., Nie S. (2019). Protective effects of β-glucan isolated from highland barley on ethanol-induced gastric damage in rats and its benefits to mice gut conditions. Food Res. Int..

[53] Rahaman M.M., Hossain R., Herrera-Bravo J., Islam M.T., Atolani O., Adeyemi O.S., Owolodun O.A., Kambizi L., Daştan S.D., Calina D. (2023). Natural antioxidants from some fruits, seeds, foods, natural products, and associated health benefits: An update. Food Sci. Nutr..

[54] Zhang Y.-J., Gan R.-Y., Li S., Zhou Y., Li A.-N., Xu D.-P., Li H.-B. (2015). Antioxidant phytochemicals for the prevention and treatment of chronic diseases. Molecules.

[55] Salim F., Adnan N., Shuib N.S., Yusof R.M. (2022). Antioxidants for Health Management. J. Intelek.

[56] McDermott J.H. (2000). Antioxidant nutrients: Current dietary recommendations and research update. J. Am. Pharm. Assoc..

